# Association of asthma and lung cancer risk: A pool of cohort studies and Mendelian randomization analysis

**DOI:** 10.1097/MD.0000000000035060

**Published:** 2023-02-02

**Authors:** Qinyao Huang, Yunxia Huang, Senkai Xu, Xiaojun Yuan, Xinqi Liu, Zisheng Chen

**Affiliations:** a Department of Respiratory and Critical Care Medicine, the Sixth Affiliated Hospital of Guangzhou Medical University, Qingyuan People’s Hospital, Qingyuan, China; b The Sixth Clinical College, Guangzhou Medical University, Guangzhou, China.

**Keywords:** asthma, lung cancer risk, mendelian randomization, meta-analysis

## Abstract

**Background:**

Over the past 2 decades, population-based studies have shown an increased association between asthma and the risk of lung cancer. However, the causal links between these 2 conditions remain poorly understood.

**Methods:**

We conducted a comprehensive search of various databases, including PubMed, Embase, Web of Science, and Cochrane Library, up until May 04, 2023. Only articles published in English were included in our study. We performed a meta-analysis using random-effects models to calculate the odds ratio (OR) and corresponding 95% confidence interval (CI). Subgroup analyses were conducted based on study design, gender, and histologic types. We also conducted a 2-sample Mendelian randomization (MR) using the genome-wide association study pooled data (408,422 people) published by the UK Biobank to explore further the potential causal relationship between asthma and lung cancer.

**Results:**

Our meta-analysis reviewed 24 population-based cohort studies involving 1072,502 patients, revealing that asthma is significantly associated with an increased risk of lung cancer (OR = 1.29, 95% CI 1.19–1.38) in all individuals. Subgroup analysis showed a significantly higher risk of lung cancer in females with asthma (OR = 1.23, 95% CI 1.01–1.49). We found no significant association between asthma and lung adenocarcinoma (LUAD) (OR = 0.76, 95% CI 0.54–1.05), lung squamous carcinomas (LUSC) (OR = 1.09, 95% CI 0.79–1.50), or small-cell lung cancer (SCLC) (OR = 1.00, 95% CI 0.68–1.49). Interestingly, our MR analysis supported an increasing causality between asthma and lung cancer (OR = 1.11, 95% CI 1.04–1.17, *P* = .0008), specifically in those who ever smoker (OR = 1.09, 95% CI 1.01–1.16, *P* = .0173) and LUSC pathological type (OR = 1.15, 95% CI 1.05–1.26, *P* = .0038).

**Conclusion:**

Through meta-analysis, our study confirms that patients with asthma have a higher risk of developing lung cancer. Our MR study further support an increasing causal relationship between asthma and the risk of lung cancer, particularly in smokers and LUSC. Future studies examining the link between asthma and the risk of developing lung cancer should consider the bias of controlled and uncontrolled asthma.

## 1. Introduction

Asthma is a common chronic, noncommunicable lung disease that affects approximately 334 million people globally, with a global prevalence in adults of 4.3% (95% CI 4.2–4.4).^[1]^ The condition is marked by persistent inflammation, reversible airway obstruction and increased bronchial reactivity. These symptoms can cause wheezing, coughing, chest tightness, and shortness of breath.^[1,2]^ According to recent studies, asthma is caused by a combination of Th1 (aiding in eliminating the pathogen but involving in airway inflammation), Th2 (causing bronchoconstriction), and Th17 (inducing asthma airway remodeling) immunologic mechanisms and genetic predisposition (related gene: ADAM33, PHF11, DPP10, GPRA, and SPINK5).^[3]^

In recent decades, several studies have explored the link between asthma and lung cancer. Presently, the antigenic stimulation theory is widely supported, suggesting that the continuous inflammatory state of the lungs in patients with asthma can result in oxidative damage, thus raising the likelihood of lung cancer.^[4–6]^ However, some studies propose the enhanced immune surveillance theory, which suggests that asthma may reduce the risk of lung cancer by increasing the clearance rate of toxins and carcinogens from the bronchoalveolar epithelium and stimulating the regeneration of cells to recover inflammatory lung tissue.^[7–10]^

Lung cancer is the second leading cause of years of life lost due to premature mortality.^[11]^ In the world, for both sexes combined, lung cancer accounts for 11.4% of the total cases and causes 18.0% of the total cancer deaths, meaning an estimated 2.2 million new cancer cases and 1.8 million deaths in 2020; While, in Western Europe, the lung cancer incidence ratio of male is 41.7% and female is 25.0%.^[12]^ Two meta-analyses have suggested that asthma significantly increases the risk of lung cancer,^[13,14]^ while others have disagreed with this conclusion.^[15–17]^ Therefore, the relationship and causality between asthma and lung cancer remain uncertain.

To fully comprehend the connection between asthma and the risk of lung cancer, we looked at pertinent cohort studies. However, this meta-analysis limitations include a lack of data supporting the causal link between asthma and lung cancer and other confounding variables like BMI, sinusitis, systemic eosinophilia, and elevated PM2.5 air pollution. These factors not only aggravate asthma but also have a direct link to lung cancer. Mendelian randomization (MR) is another approach to infer potential causal effects of exposure on outcomes by using genetic variants as instrumental variables.^[18]^ Since genetic variants are randomly assigned at meiosis and fertilization, they are relatively independent of self-selected behavior and are established before disease occurs, thus minimizing the problem of confounding and reverse causality.^[19]^

**Figure 1.**
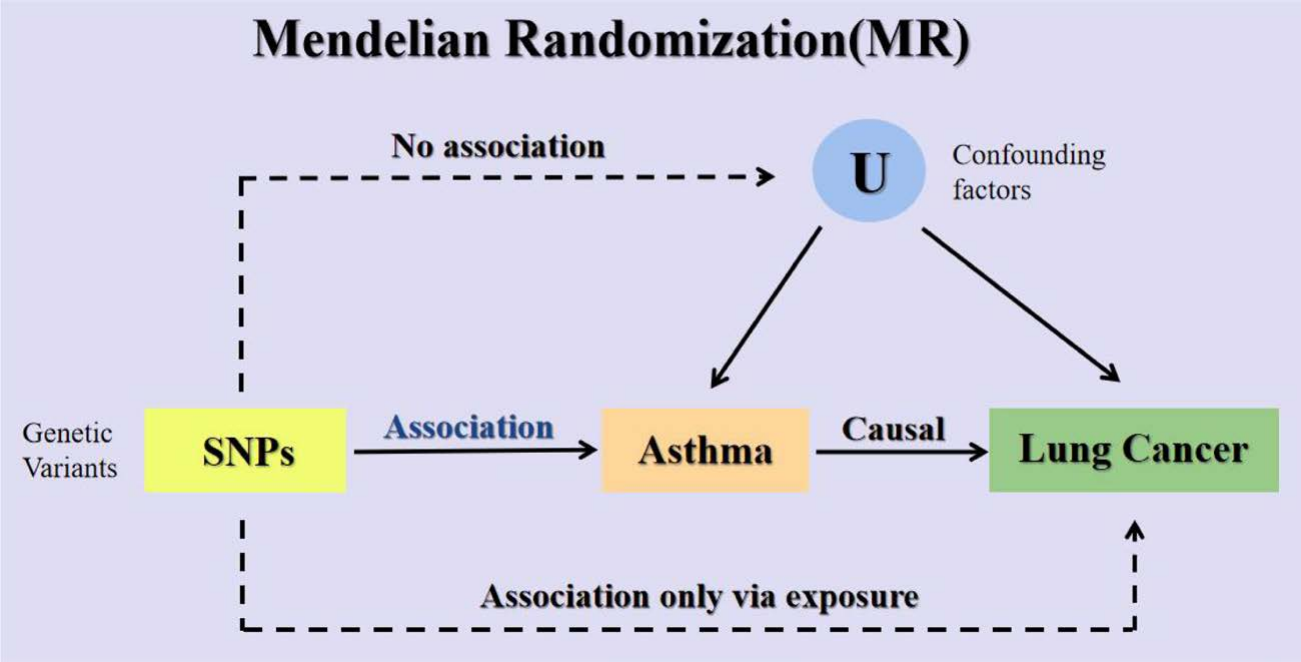
Illustrative diagram of Mendelian randomization assumptions.

To determine whether genetic susceptibility to asthma is causally related to lung cancer and its pathological subtypes, we conducted a 2-sample MR analysis. The MR approach relies on 3 key assumptions, as illustrated in Figure 1. Firstly, genetic variation must be strongly correlated with exposure (in this case, asthma). Secondly, genetic variation must be independent of any confounding factors associated with the outcome (in this case, lung cancer). Finally, genetic variation must affect the outcome only through exposure and not through other pathways (also known as the absence of horizontal pleiotropy).^[20]^ This approach typically uses the largest available genome-wide association studies (GWAS) pooled statistics from published data, summarized in Supplementary Materials Table 1, http://links.lww.com/MD/K514.

## 2. Materials and methods

### 2.1. Literature search

Our analysis included prospective cohort and case-control studies that reported at least one risk factor-outcome combination. A systematic search was conducted using PubMed, Embase, Web of Science, and Cochrane Library databases until May 04, 2023, and only articles published in English were included. Keywords used included “lung cancer,” “asthma,” “risk,” “incidence,” “cohort,” “case-control,” and their corresponding medical subject headings terms (Supplementary materials Table 2, http://links.lww.com/MD/K515). Articles were included if they met the following criteria: included both prospective cohort studies and case-control studies, involved patients with confirmed asthma, measured the risk of lung cancer among patients with confirmed asthma or without (odds ratio (OR)/relative risk (RR)/hazard ratio (HR) with corresponding 95% CI of lung cancer incidence), and were written in English. Articles were excluded if they met any of the following exclusion criteria: OR/RR/HR with corresponding 95% CI could not be obtained or estimated from the study, poor quality, individual cases, non-availability of data, or literature without the above outcome indicators. Our research was registered in the Prospective Register of Systematic Reviews (PROSPERO ID CRD42022363745). These data are publicly available, so no further ethical approval was required for this work.

### 2.2. Data acquisition

Data were extracted independently by 4 investigators (Y.H., S.X., X.L., Q.H., X.Y.), and a consensus was reached between 4 reviewers in disputes between them. The senior investigator reviewed the results (Z.C.). Information regarding target outcomes was obtained and contained in WPS software when available. General data were recorded from each study: the first author, year of publication, sample size, sample inclusion criteria, the age of the subjects, sex, intervention, and measurement indicators of the study subjects etc. The outcome of incidence was obtained to compare asthma and without-asthma groups.

### 2.3. Quality assessment

Prospective cohort and case-control studies were analyzed using the Newcastle-Ottawa Scale (NOS) to measure bias. Those studies with a performance score of at least 7 were considered high-quality. The studies were assessed independently by 2 investigators (X.L., X.Y.). The third investigator (Q.H.) was notified of any divergences generated during this process, resulting in a consensus being reached.

### 2.4. Statistical analyses in meta-analysis

Differences in RR, OR, HR, and other metrics can be disregarded if it is assumed that the results of an included study were relatively constrained across all populations and subgroups considered. To combine the data, we gathered study-specific OR/HR/RR and changed them to OR with a corresponding 95% CI for lung cancer. We looked into study heterogeneity using the I^2^ statistic and Cochran Q test. The I^2^ statistic was deemed significant at 50% for statistical heterogeneity. A random-effects model was applied when the I^2^ was >50% and the *P* value was <0.5. Otherwise, a fixed-effects model was used. A gender-based stratified analysis was performed using the data. A subgroup analysis of the various types of lung cancer was also considered. The Funnel plot and Begg and Egger tests were applied to examine publication bias. Each study was eliminated one at a time for sensitivity analysis. Stata software was used for all statistical manipulation (V.11, StataCorp, TX). The 0.05 cutoff was chosen as the statistical significance level.

### 2.5. Mendelian randomization analysis

#### 2.5.1. Study design.

Figure 1 illustrates the 3 key assumptions of MR analysis: (i) the relevance assumption, which requires a strong association between the instrumental variables and the exposure of interest; (ii) the independence assumption, which assumes no common cause shared with the outcome; and (iii) the exclusion restriction assumption, which posits that the SNPs influence the outcome solely through the exposure pathway.

#### 2.5.2. Data source.

For our MR analyses, we utilized summary-level data from the most extensive, publicly accessible GWAS for each trait (see Supplementary materials Table 1, http://links.lww.com/MD/K514). Specifically, we obtained summary-level data for asthma from the UK Biobank study (56,167 cases and 352,255 controls of European descent).^[21]^ A summary of lung cancer risk statistics, which includes estimates of odds ratios and standard errors for instrumental SNPs, can be found in the Transdisciplinary Lung Cancer Study (TRICL). These statistics are derived from a GWAS pooled analysis of 29,266 lung cancer cases and 56,450 controls.^[22]^ The original publications can find details regarding recruitment procedures and diagnostic criteria.^[22]^ All cases and controls included in these studies were of European descent, and there was no significant overlap between the populations analyzed in the GWAS.

#### 2.5.3. Genetic instrument selection.

By grouping SNPs in the 10,000-kilobase window of the 1000 Genomes European reference panel at the threshold of linkage disequilibrium (LD) r2 > 0.001, we were able to obtain independent genetic variants reaching genome-wide significance (p 5*10–8). The effects of SNPs on exposure and outcome were then synchronized to ensure that the values were signed to the same alleles. After data harmonization, we eliminated palindromic SNPs with intermediate allele frequencies (>0.42).

#### 2.5.4. Quality control of IVs.

We performed heterogeneity tests to identify outliers and correct them to increase the precision and sturdiness of genetic instruments. When horizontal pleiotropy effects were present, we used Cochran Q test for the IVW model fitting^[23]^ and Rucker Q’ test for the MR-Egger model fitting.^[24]^ We calculated the modified Q and Q’ tests and eliminated outliers with a nominal significance level of 0.05 using the “ivw radial” (alpha = 0.05, weights = 1, tol = 0.0001) and “egger radial” (alpha = 0.05, weights = 1) functions in the RadialMR v0.4R package (https://github.com/WSpiller/ RadialMR/).^[25]^ Radial variants of IVW automatically detected outliers.

The *F* statistic is a measure of instrument strength that is related to the proportion of variance in the phenotype explained by the genetic variants (*R*2), sample size (*N*) and the number of instruments (*k*) by the formula *F* = *R*2 (*N* − *k* − 1)/*k*(1 − *R*2).^[26]^ To calculate the Ri 2 for instrument i, we approximate using the formula Ri 2 = 2 × EAFi × (1 − EAFi)×βi 2, with EAFi representing the effect allele frequency and βi representing the estimated genetic effect on exposure. An F statistic of ≥ 10 indicates a relatively low risk of weak instrument bias in MR analysis.^[27]^

### 2.6. Statistical analyses

To explore the causal links between asthma and lung cancer, we employed 2-sample MR analyses. Our primary causal inference relied on an IVW regression using a multiplicative random effects model. However, the IVW results can be distorted if any SNPs exhibit horizontal pleiotropy.^[28]^ Therefore, we also used the weighted median,^[29]^ weighted mode,^[30]^ MR-egger,^[31]^ and MR-Robust Adjusted Profile Score (RAPS)^[32]^ methods based on different assumptions (Supplementary materials Table 3, http://links.lww.com/MD/K516) to make our results robustly keyed, as including multivariate instrumental variables can lead to bias in the IVW estimates.^[28]^

We also employed the fixed-effect variance weighted analysis, where Cochran Q statistic was utilized to assess the heterogeneity caused by different genetic variants, with *P* < .05 indicating the presence of pleiotropy.^[33]^ In instances where Cochran Q signaled potential pleiotropy, we employed a random-effects IVW MR analysis. We utilized MR-Egger regression to explore the possibility of horizontal pleiotropy and assessed the intercept term, where *P* < .05 indicated directional pleiotropic bias.^[31]^ In the presence of horizontal pleiotropy, the slope coefficient from the MR-Egger regression offered a reliable estimate of the causal effect. By comparing the observed distance of all variants from the regression line (i.e., the residual sum of squares) with the anticipated distance under the null hypothesis of no horizontal pleiotropy, we also used MR-PRESSO to assess the presence of pleiotropy.^[34]^ Leave-one-out (LOO) analysis was performed to assess the influence of individual variations on the observed associations. We further estimated asthma potential reverse causal effect on lung cancer and its subtypes by performing the MR Steiger test to estimate.^[35]^ We calculated the statistical power using the method described by Brion et al^[36]^

(https://shiny.cnsgenomics.com/mRnd/). A sufficient power of over 80% was recommended.

## 3. Results

### 3.1. Search results and study characteristics

**Figure 2.**
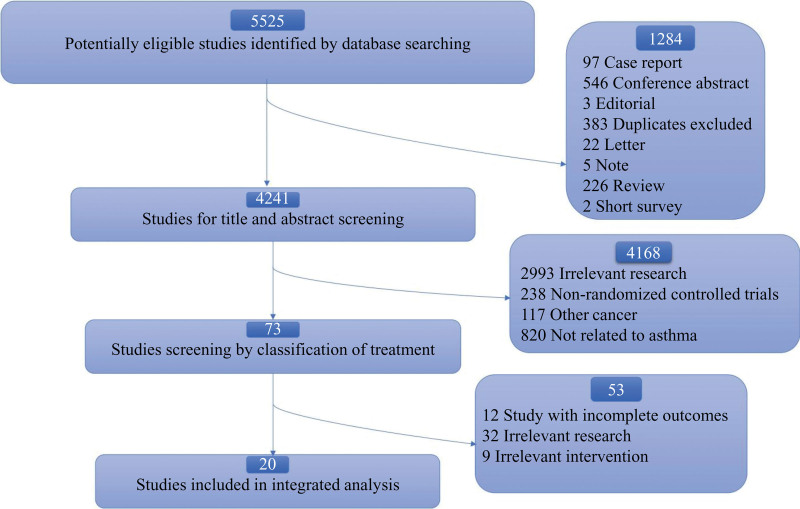
Flow diagram outlining the search strategy and study identification for meta-analysis.

The selection process flowchart is shown in Figure 2. According to the search strategy that is the network mentioned above databases, 5525 potentially eligible studies were identified. After a conscientious comparison of the relevant research, 4241 studies were considered, while 1284 were excluded (97 case reports, 546 conference abstracts, 3 editorials, 383 duplicates, 22 letters, 5 notes, 226 reviews, and 2 short surveys). Then, based on studies for the title and abstract screening, 73 articles were included, and 4168 were ruled out (2993 irrelevant studies, 238 non-randomized controlled trials, 117 not related to lung cancer, 820 not related to asthma). Finally, 20 articles remained after reading the full articles, and 53 were removed (12 studies with incomplete outcomes, 32 irrelevant studies, and 9 irrelevant interventions). We also considered the relevant investigations by the International Lung Cancer Consortium. Among them, Agnihotram V. Ramanakumar article included 3 independent cohort studies, so these were recorded as 22 studies.

The characteristics of these studies are shown in Table 1. 22 studies based on different races were from developed and developing countries, from which 7 were cohort studies, and 15 were case-control studies. In 20 articles, 1072,502 participants participated in the relevant research, from which 914,146 people were without asthma; 158,356 belonged to the asthma group over 1.23 years to 22 years. There was a male ratio between 0% and 100% because some articles only investigated men or women, and the mean age ranged from 39.6 (±13.88) to 70.55 (±8.80) years.^[2,6,7,37,40,42,44,45,48–53]^ In addition, the mean BMI of the no asthma group varied from 23.7 (±3.6) kg/m^2^ to 29.4 (±5.45) kg/m^2^. The mean BMI of the asthma group ranged from 23.7 (±3.6) kg/m^2^ to 31.23 (±5.64) kg/m^2^, which showed no significant difference with the no asthma group. What more, only 2 studies mentioned the proportion of family history of asthma, which was between 22.77% and 65.33% in the no asthma group, while between 18.75% and 65.33% in the asthma group,^[45,48]^ from which, there wasn’t any obvious difference. Finally, one study mentioned 7.24% of patients in the no asthma group suffered from allergic rhinitis as opposed to 56.65% in the asthma group.^[52]^ Meanwhile, one study noted that 8363 patients used inhaled corticosteroids, and 5660 utilized oral corticoid.^[2]^

**Table 1:** Characteristics of the included studies in the meta-analysis.

Study	Country	Race	Study design	Total number of samples	Male (%)	Age at baseline	Total length of follow up (years)	No asthma group sample			Asthma group sample			BMI (kg/m^*2*^)				Allergic rhinitis (%)								Family history of asthma (%)								Hormone		
														No asthma		Asthma		No asthma				Asthma				No asthma				Asthma						
								Total	Male	Female	Total	Male	Female	Mean	SD	Mean	SD	Yes		No		Yes		No		Yes		No		Yes		No		No	Inhaled corticosteroids	Oral corticoid
																		Sample	%	Sample	%	Sample	%	Sample	%	Sample	%	Sample	%	Sample	%	Sample	%			
Alavanja et al.^[37]^	America	White	Case–control study	1348	0	70.55 (8.80)	5	1272	0	1272	76	0	76	NA	NA	NA	NA	NA	NA	NA	NA	NA	NA	NA	NA	NA	NA	NA	NA	NA	NA	NA	NA	NA	NA	NA
Wu et al.^[38]^	America	Yellow/black/white/other	Case–control study	1633	0	NA	5	1505	0	1505	128	0	128	NA	NA	NA	NA	NA	NA	NA	NA	NA	NA	NA	NA	NA	NA	NA	NA	NA	NA	NA	NA	NA	NA	NA
Mayne et al.^[39]^	America	NA	Case–control study	873	50.11	67.60 (1.11)	2	832	NA	NA	41	NA	NA	NA	NA	NA	NA	NA	NA	NA	NA	NA	NA	NA	NA	NA	NA	NA	NA	NA	NA	NA	NA	NA	NA	NA
Brownson et al.^[40]^	America	NA	Case–control study	105	0	66.30 (10.05)	2	39	0	39	66	0	66	NA	NA	NA	NA	NA	NA	NA	NA	NA	NA	NA	NA	NA	NA	NA	NA	NA	NA	NA	NA	NA	NA	NA
Osann et al.^[41]^	America	White	Case–control study	302	0	62.31	3	204	0	204	98	0	98	NA	NA	NA	NA	NA	NA	NA	NA	NA	NA	NA	NA	NA	NA	NA	NA	NA	NA	NA	NA	NA	NA	NA
Brenner et al.^[42]^	China	Yellow	Case–control study	2644	74.13	55.98 (9.23)	4	2507	2072	435	137	102	35	NA	NA	NA	NA	NA	NA	NA	NA	NA	NA	NA	NA	NA	NA	NA	NA	NA	NA	NA	NA	NA	NA	NA
Talbot-Smith et al.^[43]^	Australia	NA	Cohort study	2711	48.87	50.32 (16.65)	18	2432	1201	1231	279	124	155	25.41	3.91	25.41	3.91	NA	NA	NA	NA	NA	NA	NA	NA	NA	NA	NA	NA	NA	NA	NA	NA	NA	NA	NA
Littman et al.^[44]^	America	Black/ white/ other/unknown	Cohort study	15600	65.4	57.42 (7.25)	17	14205	9290	4915	1395	778	617	27.39	2.81	27.39	2.81	NA	NA	NA	NA	NA	NA	NA	NA	NA	NA	NA	NA	NA	NA	NA	NA	NA	NA	NA
González-Pérez et al.^[2]^	Britain	NA	Case–control study	19658	43.27	46.12 (15.59)	8	9002	3,865	5,137	10,656	4648	6008	NA	NA	NA	NA	NA	NA	NA	NA	NA	NA	NA	NA	NA	NA	NA	NA	NA	NA	NA	NA	NA	8363	5660
Gorlova et al.^[45]^	America	White/hispanic/black	Case–control study	372	29.87	60.98 (11.92)	8	337	101	236	35	11	24	27.43	5.57	27.43	5.57	NA	NA	NA	NA	NA	NA	NA	NA	220	65.33	117	34.67	23	65.33	12	34.67	NA	NA	NA
Wang et al.^[46]^	Germany	White	Case–control study	3285	NA	61.89 (6.62)	3	3052	NA	NA	233	NA	NA	27.59	4.33	27.59	4.33	NA	NA	NA	NA	NA	NA	NA	NA	NA	NA	NA	NA	NA	NA	NA	NA	NA	NA	NA
Ramanakumar et al.^[47]^ (Study I)	Canada	White	Case–control study	1267	100	58.20 (8.43)	7	512	512	0	755	0	755	NA	NA	NA	NA	NA	NA	NA	NA	NA	NA	NA	NA	NA	NA	NA	NA	NA	NA	NA	NA	NA	NA	NA
Ramanakumar et al.^[47]^ (Study II)	Canada	White	Case–control study	1664	100	63.22 (8.97)	6	925	925	0	739	739	0	NA	NA	NA	NA	NA	NA	NA	NA	NA	NA	NA	NA	NA	NA	NA	NA	NA	NA	NA	NA	NA	NA	NA
Ramanakumar et al.^[47]^ (Study III)	Canada	White	Case–control study	1082	0	60.25 (10.02)	6	616	0	616	466	0	466	NA	NA	NA	NA	NA	NA	NA	NA	NA	NA	NA	NA	NA	NA	NA	NA	NA	NA	NA	NA	NA	NA	NA
Liang et al.^[48]^	China	Yellow	Case–control study	442	0	54.63 (11.43)	5	426	0	426	16	0	16	23.7	3.6	23.7	3.6	NA	NA	NA	NA	NA	NA	NA	NA	97	22.77	329	77.23	3	18.75	13	81.25	NA	NA	NA
Koshiol et al.^[49]^	Italy	White	Case–control study	4018	77.6	66.08 (4.31)	3	3841	2981	860	177	137	40	NA	NA	NA	NA	NA	NA	NA	NA	NA	NA	NA	NA	NA	NA	NA	NA	NA	NA	NA	NA	NA	NA	NA
El-Zein et al.^[7]^	Canada	White/other	Case–control study	2655	59.92	63.41 (8.53)	6	2387	1460	927	268	131	137	NA	NA	NA	NA	NA	NA	NA	NA	NA	NA	NA	NA	NA	NA	NA	NA	NA	NA	NA	NA	NA	NA	NA
Kantor et al.^[50]^	America	Black/ white/ other	Cohort study	64135	40.97	51.69 (7.95)	9	54719	23470	31249	9416	2808	6608	29.4	5.45	31.23	5.64	NA	NA	NA	NA	NA	NA	NA	NA	NA	NA	NA	NA	NA	NA	NA	NA	NA	NA	NA
Woo et al.^[51]^	Korea	Yellow	Cohort study	75307	57.83	49.28 (11.88)	7.61(3.58)	68422	39580	28842	6885	3972	2913	24.23	2.71	24.22	0.07	NA	NA	NA	NA	NA	NA	NA	NA	NA	NA	NA	NA	NA	NA	NA	NA	NA	NA	NA
Jiang et al.^[52]^	Norway	NA	Cohort study	62791	46.92	49.52 (17.02)	22	59591	27960	31631	3200	1501	1699	26.3	4.0	27.16	4.75	3278	7.24	42012	92.76	1554	56.65	1189	43.35	NA	NA	NA	NA	NA	NA	NA	NA	NA	NA	NA
He et al.^[6]^	Britain	Yellow/black/mixed/white/other	Cohort study	450526	46	56.38 (8.10)	13	417257	191938	225319	33269	15304	17965	28.5	5.5	27.3	4.7	NA	NA	NA	NA	NA	NA	NA	NA	NA	NA	NA	NA	NA	NA	NA	NA	NA	NA	NA
Guo et al.^[53]^	America	Black/ white/ other	Cohort study	360084	29.56	39.6 (13.88)	1.23	270063	78946	191117	90021	27481	62540	NA	NA	NA	NA	NA	NA	NA	NA	NA	NA	NA	NA	NA	NA	NA	NA	NA	NA	NA	NA	NA	NA	NA

NA, not available.

### 3.2. Quality assessment

Case-control and cohort studies were analyzed using the Newcastle-Ottawa Scale (NOS) to measure bias. Those studies with a performance score of at least 7 were considered high-quality. In our meta-analysis, 21 studies scored 7 or more were assessed as high quality,^[2,7,37–43,45–49,51]^ and only one study scored 5 as medium quality^[44]^ (Table 2).

**Table 2:** Quality assessment of the included studies in the meta-analysis.

Source	Selection	Comparability based on design and analysis	Outcome	Total
Alavanja et al.^[37]^	++++	++	++	8
Wu et al.^[38]^	++++	++	++	8
Mayne et al.^[39]^	++++	++	++	8
Brownson et al.^[40]^	++++	++	++	8
Osann et al.^[41]^	++++	++	++	8
Brenner et al.^[42]^	++++	++	++	8
Talbot-Smith et al.^[43]^	+++	++	+++	8
Littman et al.^[44]^	++	+	++	5
González-Pérez et al.^[2]^	++++	++	+++	9
Gorlova et al.^[45]^	++++	++	++	8
Wang et al.^[46]^	++++	++	++	8
Ramanakumar et al.^[47]^ (Study I)	++++	++	++	8
Ramanakumar et al.^[47]^ (Study II)	++++	++	++	8
Ramanakumar et al.^[47]^ (Study III)	++++	++	++	8
Liang et al.^[48]^	++++	++	++	8
Koshiol et al.^[49]^	++++	++	++	8
El-Zein et al.^[7]^	++++	++	++	8
Kantor et al.^[50]^	++++	++	++	8
Woo et al.^[51]^	++++	++	++	8
Jiang et al.^[52]^	++++	++	+++	9
He et al.^[6]^	++++	++	+++	9
Guo et al.^[53]^	++++	++	++	8

### 3.3. Lung cancer risk in asthma patients

During the process of conducting a pooled analysis, we exclusively considered studies encompassing both male and female participants, resulting in the inclusion of a total of 12 studies that satisfied this criterion. However, in cases where studies solely focused on either male or female subjects, we incorporated them into subgroup analysis. Asthma patients had a slightly higher risk of developing lung cancer in all relevant studies, according to the combined OR for the overall risk of lung cancer, which was 1.35 (95% CI 1.16–1.54). The 12 studies showed significant heterogeneity (Cochran Q test = 31.28, I^2^ = 64.8%, *P* = .001). A forest plot of the OR is shown in Supplementary materials Figure 1, http://links.lww.com/MD/K527. The 24 studies (Talbot-Smith et al^[43]^ and Koshiol et al^[49]^ conducted separate analyses on male and female cohorts, resulting in a total of 24 distinct studies) that provided original data were then subjected to subgroup analysis, with the results revealing that prospective cohort studies had a pooled OR of 1.37 (95% CI 1.13–1.62) (Cochran Q test = 22.98, I^2^ = 69.5%, *P* = .002) and case-control studies had a pooled OR of 1.16 (95% CI 0.91–1.40) (Cochran Q test = 44.01, I^2^ = 65.9%, *P* = .000) (Supplementary materials Fig. 2A, http://links.lww.com/MD/K528). Thus, differences in lung cancer incidence between cohort studies and case-control studies were seen, indicating potential confounding factors existed in these studies.

**Figure 3.**
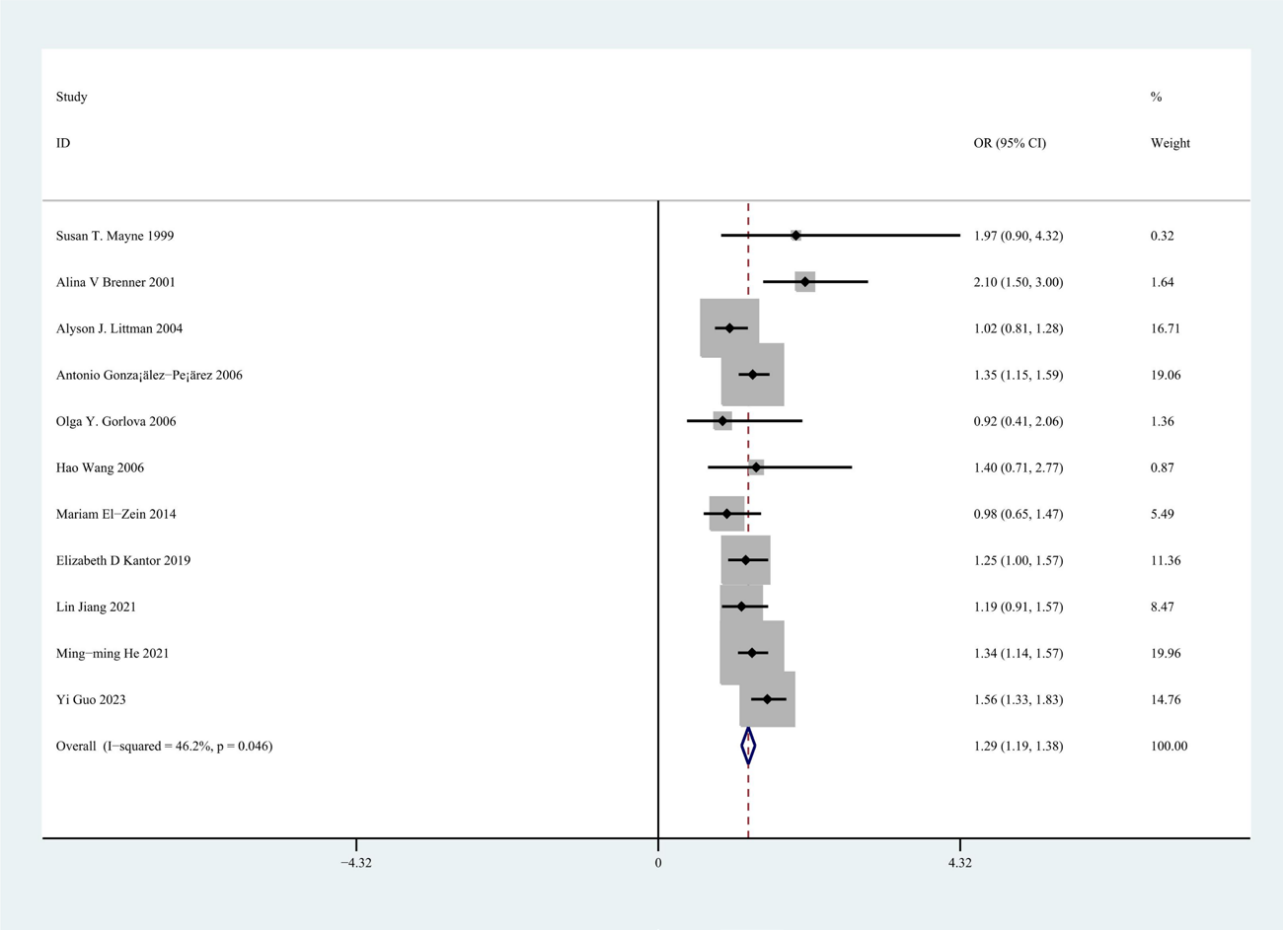
A meta-analysis of population-level cohort studies was used to compare the incidence of lung cancer in patients with and without asthma.

Throughout the sensitivity analysis, we found that the study of Woo et al^[51]^ caused the high heterogeneity, which included patients with atopic asthma (defined as asthma with one or more atopic disorders, such as allergic rhinitis or atopic dermatitis) and patients with nonatopic asthma (without any atopic disorders). Removing the study altered the pooled OR for the overall risk of lung cancer to 1.29 (95% CI 1.19–1.38), reducing heterogeneity (Cochran Q test = 18.57, I^2^ = 46.2%, *P* = .064). A forest plot of the OR is shown in Figure 3.

Based on male and female, in the 11 articles that provided original data,^[7,37–40,43,47–49]^ a subgroup analysis was conducted, resulting in a pooled OR of 0.89 (95% CI 0.70–1.12) (Cochran Q test = 9.70, I^2^ = 48.4%, *P* = .084) for males and a pooled OR of 1.23 (95%CI 1.01–1.49) (Cochran Q test = 7.11, I^2^ = 0.0%, *P* = .524) for females (Supplementary materials Fig. 2B, http://links.lww.com/MD/K528). Consequently, a significant association was observed between female asthma and an elevated risk of lung cancer, whereas male asthma did not exhibit a significant association with lung cancer.

Furthermore, there were 3 main subgroups for the pathological type of lung cancer. The subgroup analysis of lung adenocarcinoma (LUAD) suggested a pooled OR of 0.76 (95%CI 0.54–1.05) (Cochran Q test = 0.01, I^2^ = 0.0%, *P* = .939) for all people, a pooled OR of 1.28 (95% CI 0.67–2.45) (Cochran Q test = 1.71, I^2^ = 41.8%, *P* = .190) for males, and a pooled OR of 1.35 (95% CI 0.63–2.88) (Cochran Q test = 9.05, I^2^ = 77.9%, *P* = .011) for female (Supplementary materials Fig. 2C, http://links.lww.com/MD/K528). Thus, male and female asthma was not significantly associated with an increased risk of LUAD. For the subgroup analysis of LUSC, a pooled OR of 1.09 (95% CI 0.79–1.50) (Cochran Q test = 1.39, I^2^ = 27.8%, *P* = .239) was found for all people, a pooled OR of 1.13 (95% CI 0.76–1.67) (Cochran Q test = 0.04, I^2^ = 0.0%, *P* = .842) for males, and a pooled OR of 1.02 (95% CI 0.56–1.85) (Cochran Q test = 0.02, I^2^ = 0.0%, *P* = .899) for females (Supplementary materials Fig. 2D, http://links.lww.com/MD/K528). Thus, asthma was not significantly associated with an increased risk of LUSC in either male or female patients. For the subgroup analysis of SCLC, a pooled OR of 1.00 (95% CI 0.68–1.49) (Cochran Q test = 0.80, I^2^ = 0.0%, *P* = .372) was found for all people, a pooled OR of 5.52 (95% CI 1.93–15.81) (Cochran Q test = 0.06, I^2^ = 0.0%, *P* = .812) for female (Supplementary materials Fig. 2E, http://links.lww.com/MD/K528). Thus, female asthma is obviously at increased risk of SCLC.

### 3.4. Heterogeneity and publication bias

The heterogeneity significantly decreased after Woo et al^[51]^ study was removed from the equation. The meta-analysis was predicted to have no publication bias by the funnel plot, Begg test, and Egger test (*P* = .870) (Supplementary materials Fig. 3A-F, http://links.lww.com/MD/K529).

### 3.5. Sensitivity analysis

By eliminating the doubtful studies, the sensitivity analysis was finished, and the stability of the meta-analysis was evaluated. Regarding the results displayed above, there was no discernible alteration in the effects (Supplementary materials Fig. 4, http://links.lww.com/MD/K530).

### 3.6. MR results

Detailed information on the characteristics of the selected SNPs was provided in Supplementary Materials Tables 4, http://links.lww.com/MD/K517. Under the current sample size, given 12% of the phenotypic variance of asthma, our study had sufficient power (>80%) to detect an OR > 1.08 for lung cancer, lung cancer in smokers, and squamous cell lung carcinoma (Supplementary materials Table 5, http://links.lww.com/MD/K518). The range of F values was 30 to 251, meaning weak instrumental variable bias was unlikely (Supplementary materials Tables 4, http://links.lww.com/MD/K517. Outlier IVs were removed for subsequent MR analysis (Supplementary materials Tables 6, http://links.lww.com/MD/K519, Supplementary materials Fig. 5A-F, http://links.lww.com/MD/K531, The complete lists of instrumental variables used in the MR analyses are available in Supplementary Tables 7–12, http://links.lww.com/MD/K520, http://links.lww.com/MD/K521, http://links.lww.com/MD/K522, http://links.lww.com/MD/K523, http://links.lww.com/MD/K524, http://links.lww.com/MD/K525.

**Figure 4.**
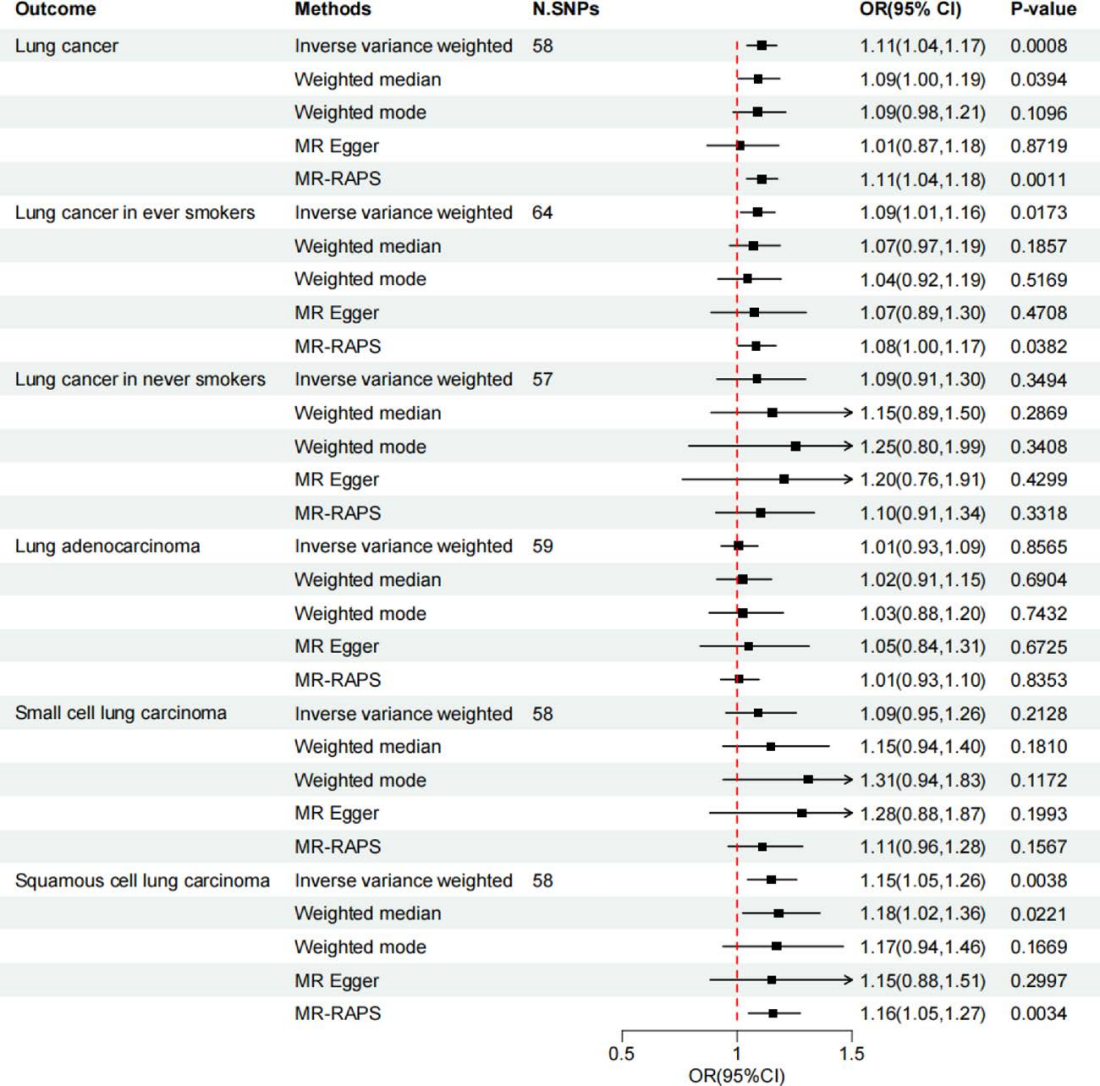
Forest plot showing the univariable MR estimates investigating the total effect of asthma on lung cancer and its subtypes. CI = confidence intervals, MR = Mendelian randomization, OR = odds ratio.

The analysis utilizing data from the UK Biobank study revealed that genetically predicted asthma was linked with an increased risk of lung cancer (IVW OR = 1.11, 95% CI 1.04–1.17, *P* = .0008). In other methodological terms, their effect values are in the same direction (Weighted median OR = 1.09,95%CI = 1.00,1.19, *P* = .0394; Weighted mode OR = 1.09, 95%CI = 0.98, 1.21, *P* = .1096; MR-egger OR = 1.01, 95%CI = 0.87, 1.18, *P* = .8719; MR-RAPS OR = 1.11, 95%CI = 1.04, 1.18, *P* = .0011) (Fig. 4). Interestingly, we found that genetically predicted asthma was associated with higher instances of lung cancer due to smoking (IVW OR = 1.09, 95%CI = 1.01, 1.16, *P* = .0173), while the effect on lung cancer due to nonsmoking was not statistically significant (IVW OR = 1.09, 95%CI = 0.91, 1.30, *P* = .3494) (Fig. 4). We also conducted MR analysis on a subset of lung cancers, which indicated a significant increase in the risk of LUSC among individuals with asthma in the UK Biobank study (OR = 1.15, 95%CI = 1.05, 1.26, *P* = .0038) (Fig. 4). However, we did not find a statistically significant effect of genetically predicted asthma on the LUAD (OR = 1.01, 95% CI 0.93–1.09, *P* = .8565) and SCLC groups (OR = 1.15, 95% CI 0.94,1.40, *P* = .181) (Fig. 4).

**Figure 5.**
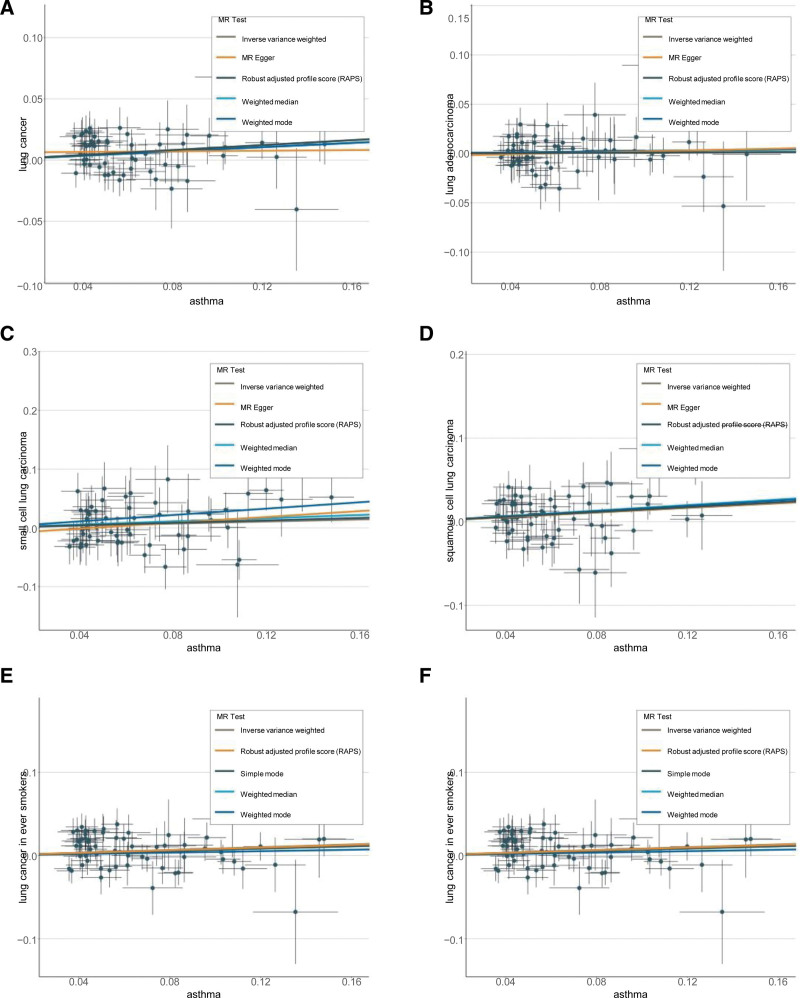
Scatter plots of the association of asthma and lung cancer and its subtypes. The slopes of each line represent the potential causal associations for each method.

Our sensitivity analysis demonstrated no indication of heterogeneity between the estimates of individual SNPs (all *P*-value > 0.05), nor did it reveal any evidence of horizontal pleiotropy (all *P*-value > 0.05). In addition, MR-PRESSO analysis did not find evidence of pleiotropy (Table 3). Figure 5A to F shows a scatter plot of the association between asthma and lung cancer and their risk subtypes, with colored lines representing the slope of the different regression analyses. Moreover, the LOO analysis showed that no single SNPS drove these results (Supplementary Fig. 6A–F, http://links.lww.com/MD/K532). We found no evidence of reverse causality across the analyses in the MR Steiger test (Supplementary materials Table 13, http://links.lww.com/MD/K526).

**Table 3:** Pleiotropy and heterogeneity test of asthma on lung cancer and its phenotypes.

Exposure	Outcomes	Heterogeneity Test				Pleiotropy test			
		IVW		MR-Egger		MR-Egger intercept			MR-PRESSO
		Q-statistics	*P*	Q-statistics	*P*	Estimate	SE	*P*	Global test P
asthma	lung cancer	48.483	.752	47.017	.769	0.006	0.005	.231	0.765
asthma	lung adenocarcinoma	34.589	.992	34.440	.990	−0.003	0.007	.701	0.992
asthma	small cell lung carcinoma	46.976	.799	46.166	.796	−0.011	0.013	.372	0.801
asthma	squamous cell lung carcinoma	56.754	.484	56.752	.447	0.000	0.008	.968	0.477
asthma	lung cancer in never smokers	52.542	.569	52.320	.539	−0.007	0.015	.639	0.561
asthma	lung cancer in ever smokers	63.033	.440	57.428	.606	0.014	0.006	.021	0.444

IVW = inverse variance-weighted, MR = mendelian randomization.

## 4. Discussion

This study combined meta-analysis and MR methods to evaluate the association between asthma and lung cancer. We provided strong evidence that asthma increases the overall risk of lung cancer using meta-analysis; subgroup analysis of 24 prior observational cohort studies (both retrospective and prospective) including 1072,502 participants supports this conclusion. Notably, MR studies also support that asthma increases the risk of lung cancer, particularly in LUSC, which is obtained in smokers. Our results are broadly robust to different MR methods that make different assumptions about horizontal polymorphism, suggesting that horizontal polymorphism is unlikely to explain our results fully.

According to the systematic literature searches, there is a contradiction between asthma and lung cancer risk based on a cohort study. Alina V Brenner et al^[42]^ indicates that asthma increases the risk of lung cancer (OR = 2.10, 95% CI 1.50–3.00). Alyson J. Littman et al^[44]^ found that asthma is not associated with lung cancer risk (OR = 1.02, 95% CI 0.81–1.26). Although prospective and retrospective cohort studies have adjusted smoking, BMI, etc, related to lung cancer risk, our study found that one important confounding factor, well controlled, partly controlled or uncontrolled among asthma patients, has not been stratified or adjusted. A previous retrospective cohort study found that asthma patients had a long diagnostic duration for lung cancer, with a mean of 36.6 years.^[54]^ Moreover, asthma severity considerably increased the likelihood of developing cancer in asthmatic individuals (HR 2.929, 95% CI 1.784–4.907).^[54]^ In addition, a prospective study of adults with partially controlled asthma had a higher risk of lung cancer (HR 1.39, 95% CI 1.00–1.92) compared with healthy groups, while no increased risk was observed in the well-controlled group (HR 0.91, 95% CI 0.54–1.52).^[52]^ Uncontrolled asthma is more prone to a decline in Forced Expiratory Volume in the first second (FEV1), similar to the Chronic Obstructive Pulmonary Disease (COPD) phenotype, and a decline in FEV1 significantly increases the risk of lung cancer.^[55]^ For the lack of studies in this field and the inconsistency of the main endpoint indicators, we cannot further evaluate the factor using a meta-analysis.

The findings of the MR analysis also support the previously well-known phenomenon that smoking hightens the risk of lung cancer, including asthma population.^[13]^ Even though patients were enrolled without regard for whether they had controlled or uncontrolled asthma, asthma medication was more standardized and better controlled in high-income areas (for example, UK Biobank) than in lower-middle-income and low-income countries.^[56]^ Inhalation of glucocorticoids and oral leukotriene receptor antagonists were the primary methods of asthma control. As is well known, LUSC predominately originates from the central airways and segmental bronchi basal cells,^[57]^ whilst the main site of asthma is in the bronchus, according to the pathogenesis of LUSC. Repeated inflammation of uncontrolled asthma stimulates the basal cell, which may increase the probability of mutation of the basal cells and raise the risk of LUSC. Previous research found that inhaled corticosteroids and oral leukotriene receptor antagonists significantly reduced lung cancer.^[58–61]^

In the future study of asthma on lung cancer risk, we believe it is necessary to divide participants into the well-controlled, partially controlled or uncontrolled group, the latter representing potentially chronic inflammation, one of the causes of cancer.^[62]^ Additionally, the lineage-tracking mouse model can also do a favor in clarifying the causal relationship between asthma and LUSC.

### 4.1. Limitations

We acknowledge that our research has certain limitations that must be considered. Firstly, we could not differentiate between controlled and uncontrolled asthma in the cohort studies for the meta-analysis, which could act as a confounding factor. Additionally, we needed more information on other influencing factors, such as PM 2.5 from the studies included, which could have impacted our results. Although we excluded potential pleiotropic SNPs and employed MR-Egger regression to minimize horizontal pleiotropy in our MR study, the possibility of pleiotropy bias cannot be entirely ruled out. Other confounding factors such as smoking, diet, alcohol consumption, chronic inflammation from infections, and occupational exposures may affect the causal links between asthma and lung cancer.^[63]^ In addition, we could not explore the effects of medication versus non-medication for asthma control on lung cancer and perform gender and ethnicity stratification analyses due to the lack of corresponding aggregated level data. The generalizability of our findings is also limited, as our study participants were primarily of European descent. Exploring the causal associations in other populations is also of great interest.

## 5. Conclusions

To summarize, our study preliminarily explores a potential causal relationship between asthma and lung cancer, with asthma exhibiting a specific association with an increased risk of lung cancer.

## Acknowledgments

The authors thank the genome-wide association study consortiums for making the MR-Base platform high-quality resources available to researchers (https://www.mrbase.org/). We acknowledge the participants and investigators of the UK Biobank. Also, We acknowledge Mike Haynes for the help of grammatical accuracy and language quality.

## Author contributions

**Data curation:** Qinyao Huang, Yunxia Huang, Senkai Xu, Xiaojun Yuan, Xinqi Liu.

**Formal analysis:** Qinyao Huang, Yunxia Huang.

**Funding acquisition:** Zisheng Chen.

**Investigation:** Qinyao Huang, Yunxia Huang, Senkai Xu, Xiaojun Yuan, Xinqi Liu, Zisheng Chen.

**Methodology:** Qinyao Huang, Yunxia Huang, Xiaojun Yuan, Xinqi Liu.

**Project administration:** Yunxia Huang.

**Software:** Qinyao Huang, Yunxia Huang.

**Supervision:** Zisheng Chen.

**Validation:** Yunxia Huang, Zisheng Chen.

**Writing – original draft:** Qinyao Huang, Yunxia Huang, Zisheng Chen.

**Writing – review & editing:** Zisheng Chen.

## Supplementary Material






































