# Supplementary figures and images for: Association of asthma and lung cancer risk: A pool of cohort studies and Mendelian randomization analysis
Source: Medicine (Baltimore). 2023 Feb 2;103(5):e35060. doi: 10.1097/MD.0000000000035060 (PMC10843492; doi:10.1097/MD.0000000000035060)

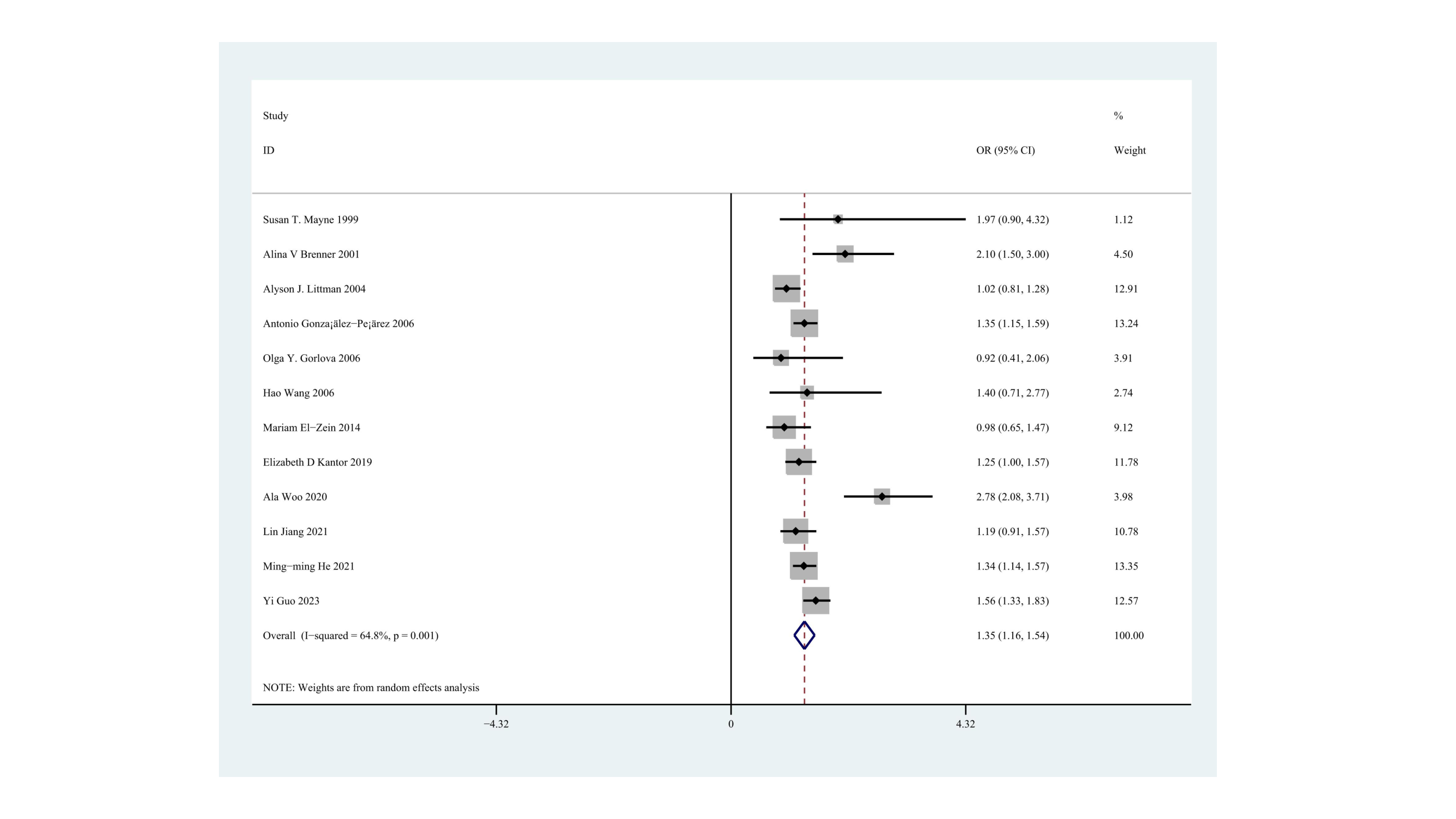

Supplement: Supplementary file 4 [file medi-103-e35060-s004.tiff]

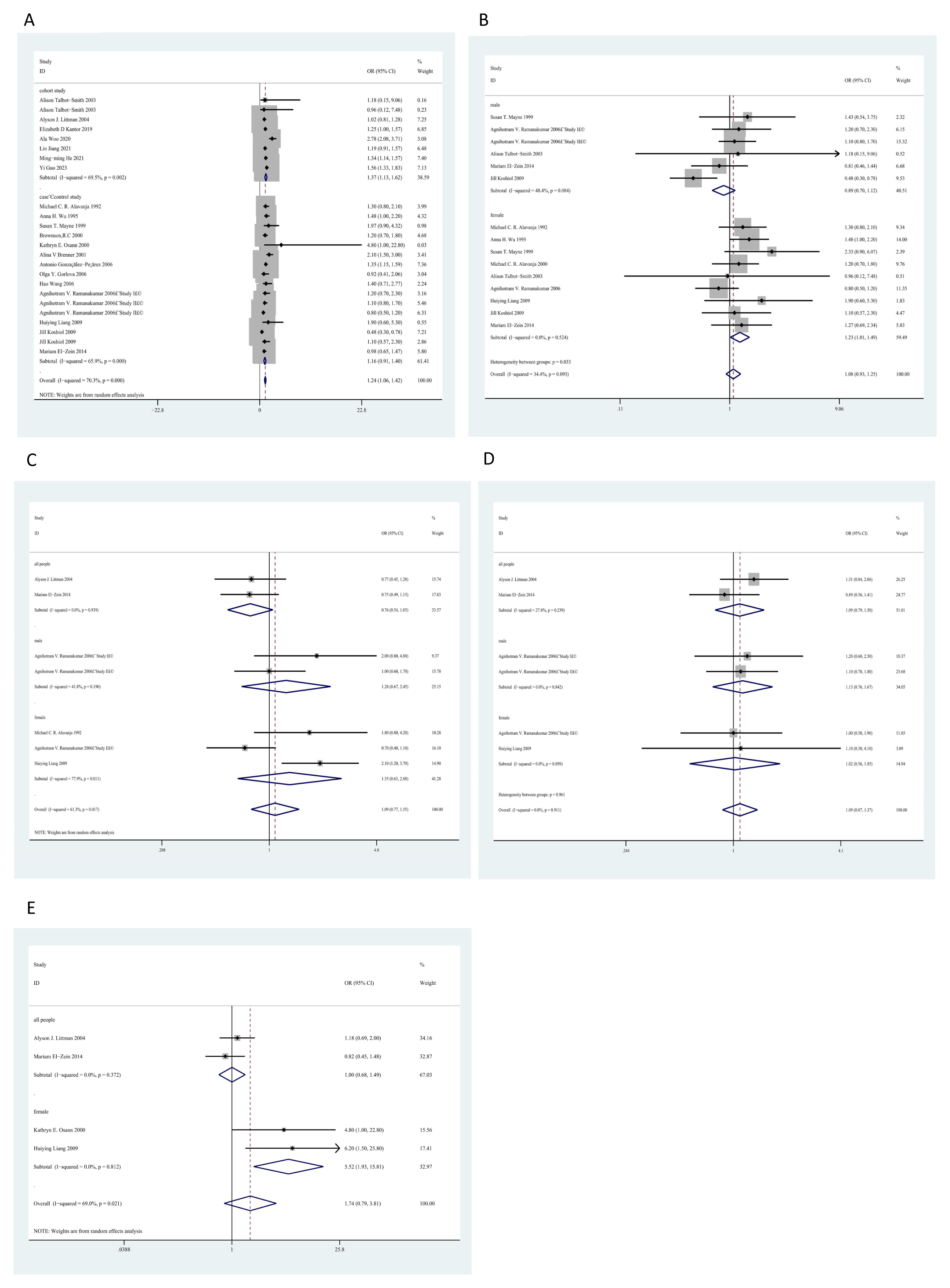

Supplement: Supplementary file 5 [file medi-103-e35060-s005.tif]

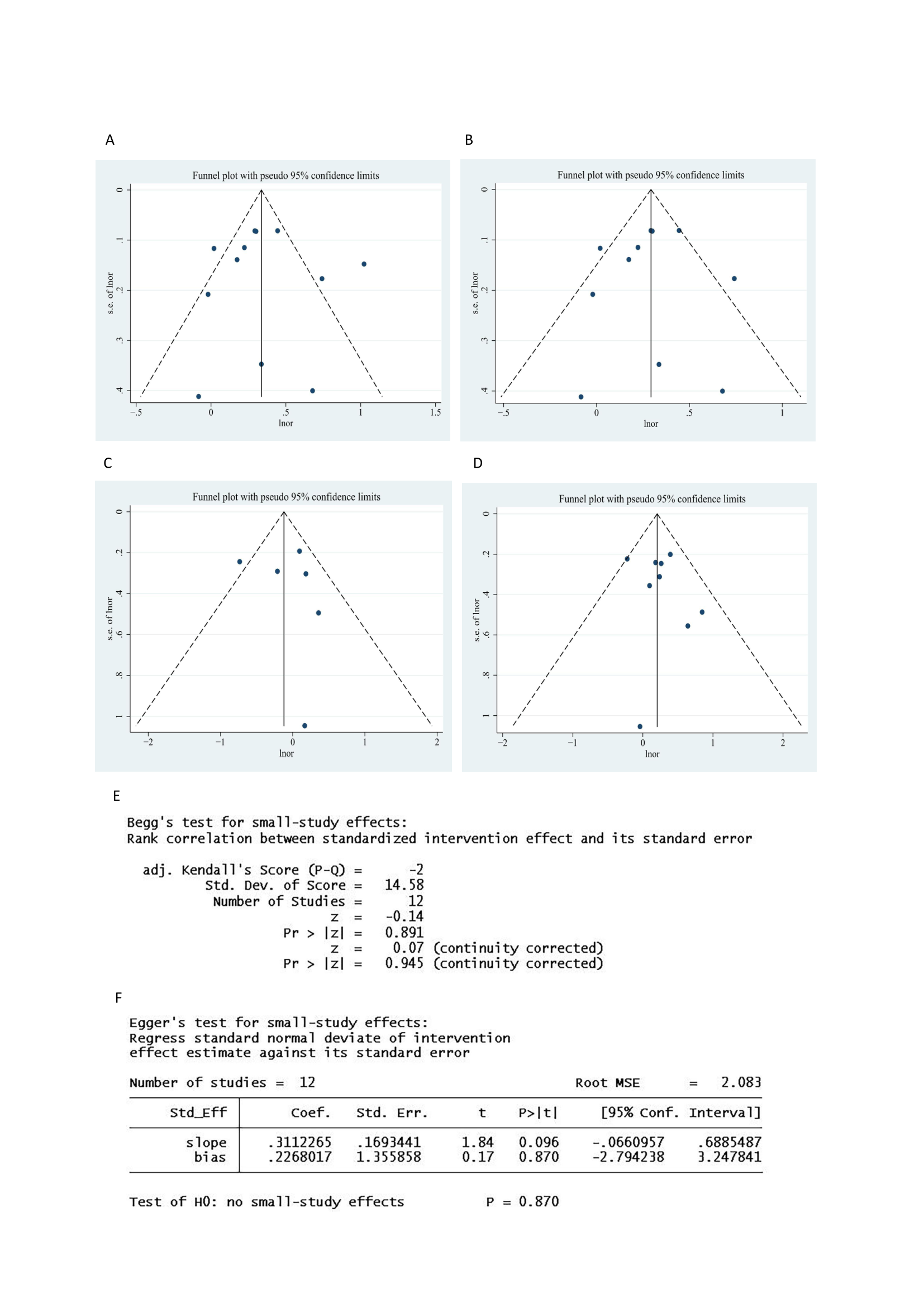

Supplement: Supplementary file 6 [file medi-103-e35060-s006.tiff]

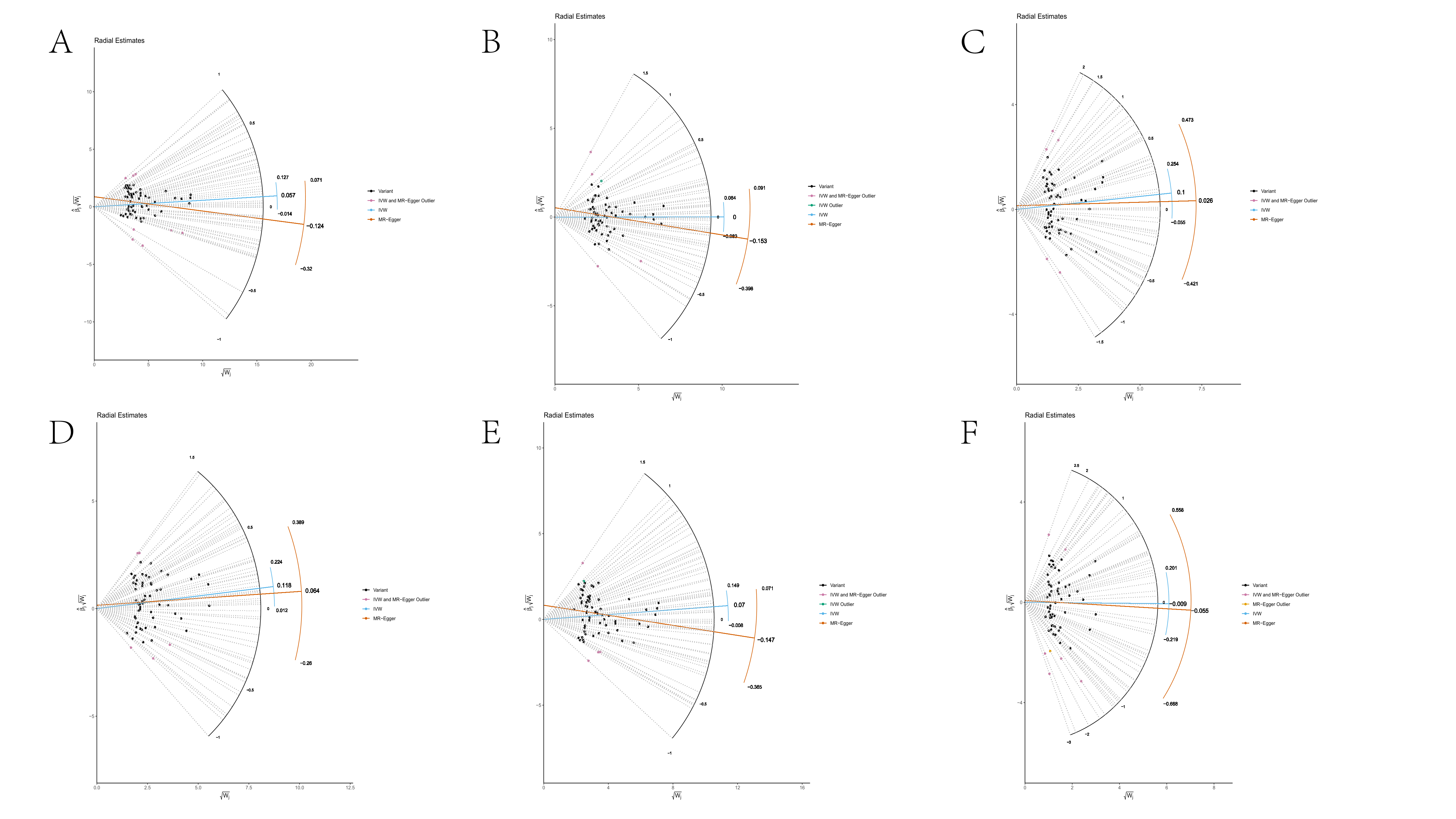

Supplement: Supplementary file 11 [file medi-103-e35060-s011.tiff]

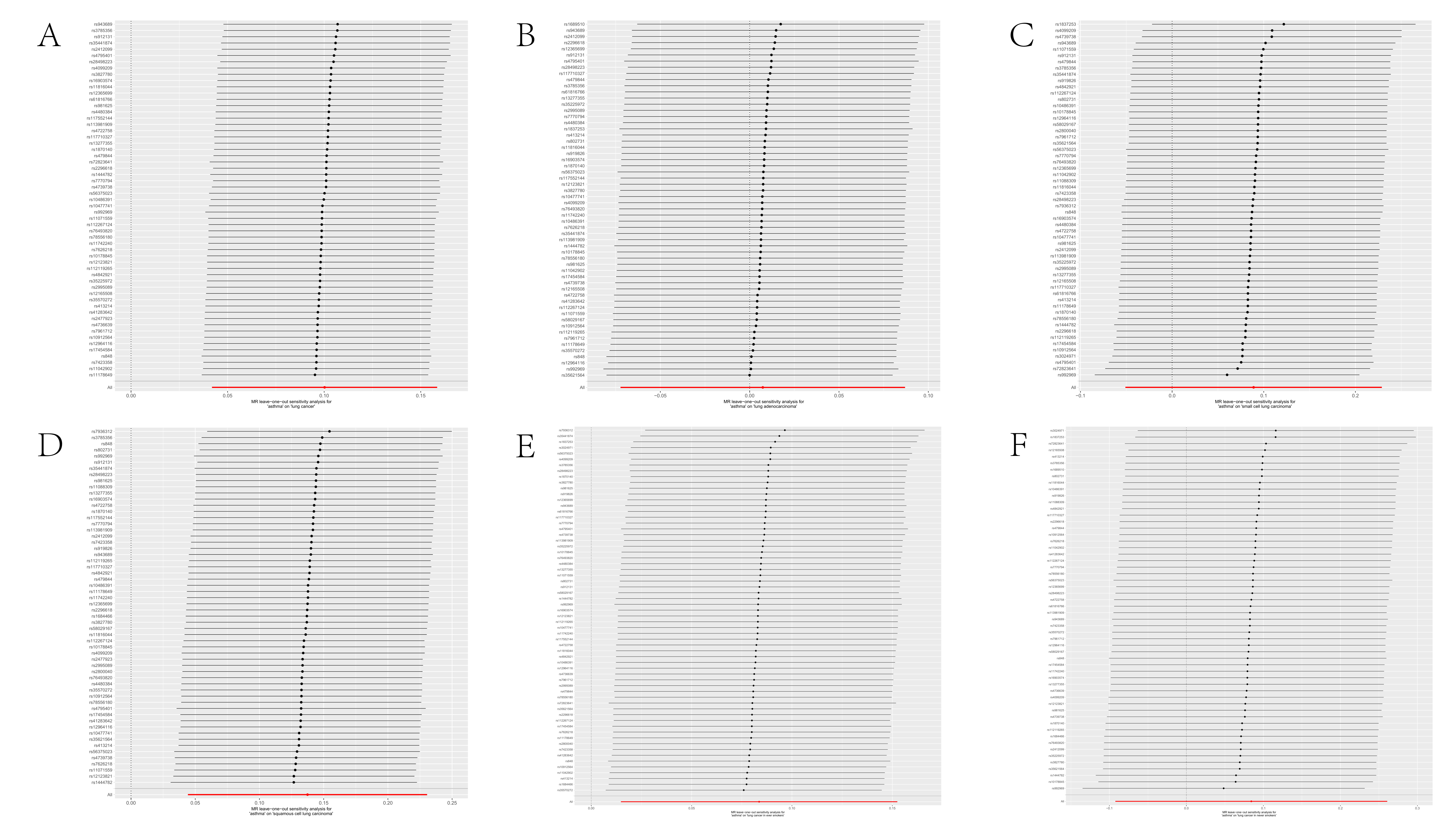

Supplement: Supplementary file 18 [file medi-103-e35060-s018.tiff]
